# Ophthalmology and Otology

**Published:** 1873-03

**Authors:** C. E. Wright

**Affiliations:** Indianapolis


### OPHTHALMOLOGY AND OTOLOGY.

BY DR. C. E. WRIGHT, OF INDIANAPOLIS.

*Eczema Aurium.*—During the process of dentition, we find eczema of the auricle in quite a number of cases, either developing in this structure primarily by extension from the surrounding parts, or in conjunction with eczema affecting the whole of the head and face. Indeed, we find it not only during dentition, but in case of debility arising from whatever cause in those children who, from their appearance, are properly supposed to be of scrofulous diathesis. A case may serve to illustrate the symptoms, and treatment of the complaint.

A child, aged fourteen months, of German parentage, well-formed and healthy, with fair hair, blue irides, was brought to me on account of eczema affecting the auricle. The left auricle was swollen and prominent; its normal elevations and depressions indistinct and not discernible in certain parts. A yellowish and bloody scab covered the greater portion of the auricle, removal of the crust showing the parts beneath red, swollen and bleeding when touched. Behind the auricle where it was attached to the head was a raw, wet fissure, secreting an offensive watery discharge, the odor of which was nearly intolerable. Separation of the sides of this fissure by drawing the auricle forwards was attended with pain. The hair in the vicinity of the ear was matted together by the secretion. On the cheek and side of the neck were separate vesicles, which, on bursting, gave rise to the peculiar yellowish scabs, some being tinged with blood. The concha and outer portion of meatus were swollen and contained a reddish, watery fluid, and some hardened crusts. The meatus, after washing out a lot of flakes of epidermis, presented a sodden appearance, as after poulticing, membrana tympani not to be discerned.

The affection had come on with the beginning of teething, and had been more or less severe ever since—sometimes better, sometimes worse than at present.

The little patient would frequently pick and rub the affected part, loosening the crusts and causing bleeding.

Owing to the irritation of swollen gums, he was sometimes quite peevish and fretful, and would then, more than at any other time, scratch and pull at his ear, but during the greater portion of the time he was apparently free from pain, and was cheerful and playful.

The right auricle was sore only behind where it was joined to the head, and was painful only when the sides of the fissure were separated, thus exposing it to the air and breaking the adhesions which had partially formed.

The child being in good health, strong and active, no constitutional treatment being indicated further than allaying the pain induced by the condition of the gums, and the itching of the excoriated parts, I ordered the following: ℞.—Potassii bromide, grs. xxxii.; syrup simple, f℥ ii.; ol. anise sem, gtt. iii. Mix. A teaspoonful occasionally for restlessness; and the following, to be applied on cotton wool to the ear: ℞.—Ol. jecoris aselli, f℥ ii.; acid tannic, ʒ i. Mix. To be applied twice daily.

The cod-liver oil is the best greasy application I have yet found for the purpose of softening and removing, or preventing the formation of the crusts of this disease, and I can recommend it as a remedy that I have fully tried and have seldom found wanting in effecting a cure.

Various washes and lotions and ointments have been recommended as external applications, and Woltsch says "it seems to be a matter of indifference as to what kind of oil or salve is used," and especially in the chronic and the impetiginous forms; yet I

have met with better success in the use of cod-liver oil, either alone or with an astringent, than with any other application.

If with these external applications we employ sedatives (such as the bromide of potassium) internally, a speedy cure is almost certain in impetiginous (the form I have usually observed accompanying dentition) eczema aurium.

Wilson (Diseases of Skin, p. 202) lays down these three rules to guide us in treating eczema aurium :

1. Eliminate, cathartics, etc.
2. Restore power, tonics, arsenic, etc.
3. Alleviate local symptoms.

Now, these rules may be useful in combatting some forms of eczema, but in cases occurring during teething, which we are more particularly considering now, the plan before indicated will generally prove successful, as it did promptly in the case related.—*Indiana Journal of Medicine.*

